# Prevalence and Morphological Investigation of Parasitic Infection in Freshwater Fish (Nile Tilapia) from Upper Egypt

**DOI:** 10.3390/ani13061088

**Published:** 2023-03-18

**Authors:** Salwa Mahmoud Abd-ELrahman, Ahmed Gareh, Hager Ibrahem Mohamed, Barakat M. Alrashdi, Ahmed Kamal Dyab, Manal F. El-Khadragy, Nady Khairy Elbarbary, Alamira Marzouk Fouad, Fatma A. El-Gohary, Ehab Kotb Elmahallawy, Sara Abdel-Aal Mohamed

**Affiliations:** 1 Department of Parasitology, Faculty of Veterinary Medicine Assiut University, Assiut 71515, Egypt; salwamahmoud@aun.edu.eg (S.M.A.-E.); hageribrahem@vet.aun.edu.eg (H.I.M.); salma@aun.edu.eg (S.A.-A.M.); 2 Department of Parasitology, Faculty of Veterinary Medicine, Aswan University, Aswan 24101, Egypt; ahmedgareh86@gmail.com; 3 Biology Department, College of Science, Jouf University, Sakaka 72388, Saudi Arabia; bmalrashdi@ju.edu.sa; 4 Department of Parasitology, Faculty of Medicine, Assiut University, Assiut 71515, Egypt; ahmed2015@aun.edu.eg; 5 Department of Biology, College of Science, Princess Nourah bint Abdulrahman University, P.O. Box 84428, Riyadh 11671, Saudi Arabia; mfelkhadragy@pnu.edu.sa; 6 Department of Food Hygiene, Faculty of Veterinary Medicine, Aswan University, Aswan 81528, Egypt; nadyvet82@yahoo.com; 7 Department of Aquatic Animal Medicine and Management, Faculty of Veterinary Medicine, Assuit University, Assiut 71526, Egypt; alamira_foad@aun.edu.eg; 8 Department of Hygiene and Zoonoses, Faculty of Veterinary Medicine, Mansoura University, Mansoura 35516, Egypt; 9 Department of Zoonoses, Faculty of Veterinary Medicine, Sohag University, Sohag 82524, Egypt

**Keywords:** Nile tilapia fish, ectoparasites, endoparasites, experimental infections, Upper Egypt

## Abstract

**Simple Summary:**

Fresh water Nile tilapia can be infected by numerous parasites, which can result in high mortality and significant economic losses. The early detection of parasites and the improved control of the major risk factors related to infection are among the main approaches for controlling infection. Limited information is available on fish parasites in Upper Egypt. The present study examined the prevalence of parasitic infection among 300 fish samples collected from different markets in Assiut Governorate, Upper Egypt, using a series of detection techniques, including microscopic, parasitological, histopathological and morphometric methods. Moreover, the associations of the demographic factors with the distribution of parasitic infection in Nile tilapia were also investigated.

**Abstract:**

Fish are a source of high-quality protein with low cholesterol, but they are susceptible to parasitic infections, which have a significant impact on aquaculture, in addition to their zoonotic potential. The present study estimated parasitic infections and evaluated the diversity of zoonotic parasites in freshwater Nile tilapia (*Oreochromis niloticus*) in Assiut Governorate, Upper Egypt. A total of 300 samples were randomly collected from the Assiut Governorate. These fish were examined for both ectoparasites and endoparasites, followed by the experimental infection of mice with encysted metacercariae (EMC) for the retrieval of the adult worms. The overall prevalence of the variable parasites was 82% (246 of 300). Both ecto- and endoparasites were detected in 41% (123 of 300) of the examined fish. The identified ectoparasites were *Gyrodactylus*, *Dactylogrus*, *Cichlidogyrus*, *Trichodina* and *Icthyophthirius multifiliis*, in 5%, 4%, 22%, 6% and 4% of the fish, respectively. The endoparasites were trematodes *(Orientocreadium batrachoides* 3%), nematodes (*Contracaecum*. 2%), acanthocephala (*Acanthosentis tilapiae* 25%) and protozoa that included *Isospora* and *Eimeria* spp., in 1% and 8% of fish, respectively. *Myxobolus* was detected in 2% of the examined fish. The overall prevalence of encysted metacercariae (EMC) was 95% (285 of 300), while infection with macroscopic EMC had a prevalence of 37% and microscopic EMC had a prevalence of 58%. The adult worms recovered from the experimental infections were *Prohemistomum vivax* and *Mesostephanus* spp., which belong to the family *Cyathocotylidae*. Collectively, these findings reflect the relatively high occurrence of parasites among the studied fish, confirming the necessity of strict measures to control infection.

## 1. Introduction

Fish are one of the main sources of animal protein in human diets [1]. Fish meat has many beneficial components, such as polyunsaturated fatty acids (PUFA), essential minerals, omega-3 and omega-6 fatty acids, antioxidants and lipid soluble vitamins, which are important for human health [2]. Nile tilapia (*Oreochromis niloticus*) is considered to be one of most common freshwater fish. In Egypt, Nile tilapia is a principal fish species inhabiting Nile River, and it is one of cheapest and most accessible fish for people. Nile tilapia possess the ability to survive depressed environmental circumstances, powerful disease resistance and low slight respiratory demands; hence, they can tolerate low oxygen and high ammonia levels [3]. However, the industrial production of Nile tilapia in Egypt is hindered by a wide range of pathogenic agents, resulting in considerable economic losses [4].

Similar to other vertebrates, fish play a significant role as the final or intermediate hosts for many parasites, with a potential for the zoonotic transmission of these parasites to humans [5]. Parasites affect the survival of fish by reducing their size, altering the behavior of infected fish and making them vulnerable to other infections, resulting in higher mortality. Therefore, parasites can cause considerable economic losses in fish production due to mortality and tissue damage [6,7]. In Egypt, there are prolonged periods of optimum warm weather that favor the multiplication of different parasites, which, in turn, affects the health of fish [8]. Fish parasites are divided into ectoparasites and endoparasites [9]. Ectoparasites on infected fish are usually identified by scratches, ulcerations of the body, hemorrhagic spots on the skin and battered fins [10]. Therefore, the ectoparasites are considered among the most hazardous groups of parasites and cause high mortality in fish [11]. Additionally, fish may be affected by many endoparasites, such as protozoa, trematodes, cestodes, nematodes and acanthocephala [12], which can cause distortions in their bodily structure and have harmful effects on the function of the affected organs [13]. Metacercaria of digenetic trematodes are considered to be one of the most important endoparasites and are responsible for great economic losses across different fish species, in terms of both fish culture and open water resources in Egypt. 

More importantly, many parasites of fish, particularly trematodes, have zoonotic importance and can seriously affect human health, leading to intestinal pain and diarrhea [14]. Humans are mainly infected through the consumption of raw or improperly cooked fish [15] containing the encysted metacercariae [16]. Recently, fish-borne zoonotic trematodes were added to the list of emerging infectious diseases according to the World Health Organization and the United Nations Food Agriculture Organization [17]. *Clonorchis sinensis*, *Opisthorchis* spp., *Heterophyes* spp., *Metagonimus* spp., *Echinostome* spp. *Nanophyetes salminicola* and *Paragonimus* spp. are considered the most important fish borne zoonotic trematodes. *Heterophyes* spp. were detected in humans in several countries of Asia and Africa [1]. The consumption of raw fish is not habitual in Egypt. Nevertheless, *H. heterophyes* have been identified by many authors, with a prevalence rate ranging between 0.1–33.8% [2,3,4,5]. Opisthorchiasis is a public health hazard in Eastern Europe and Southeast Asia [6], with more than 45 million people worldwide being infected with *Opisthorchis* spp. [7]. In addition, the Chinese liver fluke (*Clonorchis sinensis*) is the most important fish-borne zoonotic parasite in East Asia [8], and more than 15 million people are infected with *C. sinensis* worldwide [9]. Lung fluke disease (*Paragonimus* spp.) is a prevalent zoonotic trematodes in Southeast Asia, Africa and America. In Egypt and Mediterranean countries, *Herrings* and *Sardine* spp. are the most important fish species that play important roles in the transmission of disease to humans [10]. Moreover, *Diphyllobothrum* spp. and *Ligula intestinalis* are the most common zoonotic cestodes. In relation to zoonotic nematodes, *Capillaria* spp., *Contracaecum* osculatum, *Pseudoterranova decipiens*, *Eustrongylides* spp. and *Anisakis* spp. are the most common [11,12,13,14]. *Capillaria philippinensis* is another fish born zoonotic nematode [15] that has been reported by many authors in Egypt, and the disease causes serious and even lethal disease if left untreated [15,16,17].

There is very limited information available on the prevalence of parasitic infection in freshwater fish (Nile tilapia) in Upper Egypt. The present study aimed to investigate the prevalence of ectoparasitic (external) and endoparasitic (internal) infection in freshwater Nile tilapia (*Oreochromis niloticus*) in Assiut Governorate, Upper Egypt, focusing on the diversity of the zoonotic parasites that are of public health importance.

## 2. Materials and Methods

### 2.1. Ethical Considerations 

This study was approved by the research ethical committee of the Faculty of Veterinary Medicine, Assiut University, Egypt; ethical approval number: aun/vet/3/0009.

### 2.2. Study Area and Sample Collection

The current study focuses on the prevalence of different parasites in Nile tilapia (*Oreochromis niloticus*) in Assiut Governorate, located in Upper Egypt (latitude, 27°10′48.4824″ N; and longitude 31°11′21.4188″ W). A total of 300 samples (186 males and 114 females) were collected randomly from different fish markets in Assiut Governorate during the period of March 2021 to November 2021. The fish were obtained from Nile River; their weight ranged between 78 g and 127 g and their length ranged between 12 cm and 21 cm. The fish were then transported in separate labeled plastic bags to the parasitology laboratory of the Faculty of Veterinary Medicine, Assiut University, for the complete description of each fish, followed by a complete parasitological examination.

### 2.3. Examination of Fish for Different Parasites

#### 2.3.1. Macroscopic Examination

The fish were inspected externally with the naked eye and using a dissecting microscope; then, the scales were crushed carefully by a scalpel to identify any macroscopic lesions or cysts [16]. In addition, the fish were dissected at the anal opening extended anteriorly along the fish’s ventral region towards the gill chamber. In addition, another two lateral incisions were performed to reveal the body cavity, and the alimentary canal and other internal organs were examined for the presence of macroscopic adult parasites and encysted metacercariae [18].

#### 2.3.2. Microscopic Examination

Several direct smears were prepared from the body surface, fins and gills; these were examined microscopically [19]. The intestinal content was emptied into a petri dish and diluted with physiological saline; the films were then prepared and examined [18]. Adult trematodes and cestodes were collected, washed with physiological saline to remove mucus and debris, and left in a refrigerator at 4 °C until complete relaxation. Then, they were fixed according to [20] and stained in acetic acid alum carmine according to [21]. The nematodes were collected in warm 70% ethyl alcohol solution and cleaned in lactophenol for 12–24 h. They were then preserved in 70% alcohol plus 5% glycerin solution [22]. All of the prepared slides were examined to identify the parasite species, as described elsewhere [23,24,25,26,27,28,29,30,31,32,33,34,35,36,37].

#### 2.3.3. Examination of Fish Muscle by Compression Technique

The collected specimens were examined to detect any macroscopic encysted metacercariae. From each fish, a small snip of muscles from different regions of the body (head, trunk and tail regions) was compressed between two glass slides and examined microscopically. Then, the infected samples underwent artificial digestion [38].

#### 2.3.4. Artificial Tissue Digestion of Infected Samples for Isolation of Encysted Metacercariae

The infected fish muscles were cut into small portions and digested with artificial gastric juice (7.5 mg Pepsin powder, 10 mL hydrochloric acid 37%, 1000 mL distilled water). The samples were then transferred to a magnetic stirrer with a magnetic rod at 37 °C until the muscles were completely dissolved, after which the samples were sieved and allowed to settle for a few minutes. The supernatant was removed, and the sediment was aspirated and placed into Petri dishes by Pasteur pipette to collect the encysted metacercariae. The Petri dishes were placed under a dissecting binocular microscope [39,40].

#### 2.3.5. Histopathological Examination of Encysted Metacercariae

The tissue specimens from the positive samples were fixed in 10% formalin, dehydrated in ascending alcohol concentrations (50%, 70%, 90%, 95%, absolute ethanol), cleared with xylene and embedded in paraffin blocks. Then, the specimens were stained by hematoxylin and eosin and examined microscopically [41].

#### 2.3.6. Experimental Infection of Laboratory Animals with Encysted Metacercariae

The animal study and experimental infection of the laboratory animals were approved by the research ethical Committee (approval number is aun/vet/3/0009) of Faculty of Veterinary Medicine, Assiut University. To obtain the adult stage, ten albino mice (5–8 weeks old, weighted 150–300 g), free from any parasitic infection, were used. Each mouse was orally inoculated with 300 fresh metacercariae per 0.5 mL (infective dose) using a sterile syringe with a blunt gauge. Then, the infected animals were sacrificed at the seventh day post-infection under anesthesia. Their small intestines were removed, opened longitudinally and scraped with a glass slide. The samples were then washed with warm saline in Petri dishes for the detection of different parasites [42].

#### 2.3.7. Preparation of Adult Worms for Examination by Scanning Electron Microscope

Some isolated worms were washed in saline and then placed in 5% glutaraldehyde for 24–72 h. The samples were washed four times in sodium cacodylate buffer (pH 7.3) for 15 min each, followed by fixation for 2 h by adding 1% osmium tetroxide. Then, they were washed again three times with sodium cacodylate buffer (pH 7.3) and dehydrated in ascending concentrations of ethanol (i.e., 30, 50, 70, and 90%, in this order), followed by a double change of absolute ethanol for 24–48 h. The samples were cleared in xylene overnight and air dried; then, they were incubated at 20–25 °C, stocked in double Scotch tape carbon, and coated with gold. The samples were examined using SEM (Joel, JSM-5400LV Scanning Electron Microscope, Tokyo 1993, Japan) to identify the morphological characters of the isolated worms.

#### 2.3.8. Statistical Analysis 

The data were verified, coded by the researcher and analyzed using IBM-SPSS 24.0. The chi square/Monte Carlo exact test and Fisher’s exact test were used to compare the difference frequency between the altered groups (*p*-value less than 0.05 was considered significant).

## 3. Results

### 3.1. Occurrence of Fish Parasites

As presented in Table 1, the overall prevalence of all parasites was 82% (246/300) in the examined fish. Male fish tended to be more susceptible to infection, with a rate of 60%, compared to females, with a rate of 40%. The weight of the examined fish was significantly related to the rate of infection with different parasites (*p <* 0.05). There was a trend for a greater proportion of fish in winter and spring to be uninfected, but not that many more fish were infected in spring. In addition, the greatest prevalence of infection was found in the spring season, with a rate of 28.8%.

### 3.2. Prevalence of Ectoparasitic and Endoparasitic Infection in the Examined Fish

As shown in Table 2, the parasitological examination of the gills, fins and skin revealed that the overall prevalence of ectoparasites was 41% (123 of 300); the prevalence of external protozoa was 10% (*Trichodina*, 6%; *Icthyophthirius multifiliis*, 4%); the prevalence of monogenetic trematodes was 31% (*Gyrodactylus*, 5%; *Dactylogrus*, 4%; *Cichlidogyrus*, 22%). The overall prevalence of endoparasites was 41% (123 of 300), including digenetic trematodes (*Orientocreadium*, 3%), nematodes (*Contracecum*, 2%), acanthocephala (25%) and internal protozoa (*Myxobolus*, 2%; *Isospora*, 1%; *Eimeria*, 8%).

### 3.3. Prevalence of Ectoparasites in Relation to Sex, Size and Seasonal Condition in Nile Tilapia 

As shown in Table 3, the prevalence of monogenean-infected fish was 31% (93 of 300). Female fish tended to be more susceptible to infection, with a rate of 51.6%, compared to males, with a rate of 48.4%. The weight of the fish was significantly related to infection (*p* < 0.05). Infection with external monogeneans was higher in the winter season, a with rate of 35.5%. The overall prevalence of external protozoa was 10% (30 of 300), with proportionately more females infected. Fish with length 16.10 ± 0.75 cm and weight 92.70 ± 1.33 g were more susceptible to infection. The highest infection rates of external protozoa (40%) were detected in spring and summer.

### 3.4. Morphological Characterization of Ectoparasites Infecting Nile Tilapia

**Figure 1:**
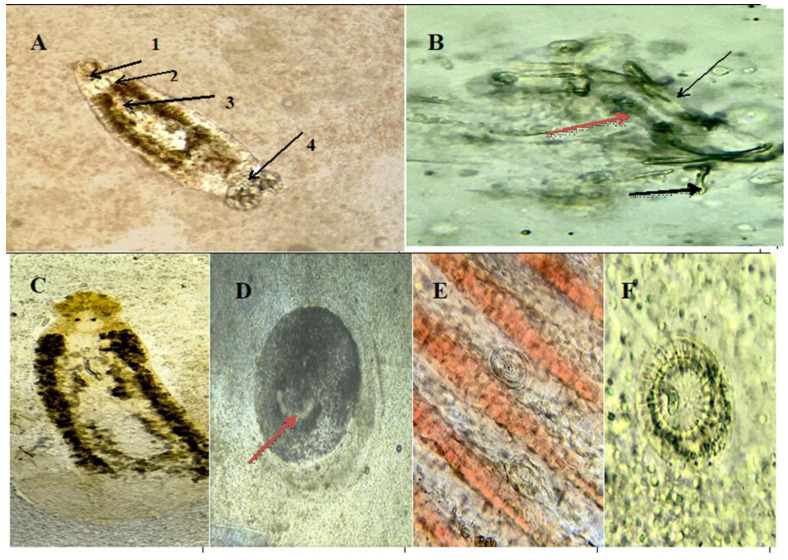
Wet mount of gill filament showing: (**A**) *Cichlidogyrus* spp. adult stage ×100 (1—eyespots, 2—pharynx, 3—copulatory organ, 4—opisthaptor); (**B**) opisthaptor of *Cichlidogyrus* spp. ×400 (red arrow shows dorsal anchor; black arrow shows ventral anchor; heavy dark arrow shows marginal hooks; (**C**) *Dactylogyrus* spp. adult stage ×100; (**D**) *Ichthyophthirius multifiliis* trophont ×1000 (arrow shows horseshoe-shaped nucleus); (**E**) *Trichodina* spp. ×100; (**F**) *Trichodina* sp. ×400.

As depicted in Figure 1, the ectoparasites were morphologically identified. *Cichlidogyrus* spp. are characterized by the presence of two pairs of eye spots and two pairs of anchors (one dorsal and one ventral). *Dactylogyrus* spp. have two pairs of eye spots, one pair of anchors and one transverse bar. *Ichthyophthirius multifiliis* have a spherical shape and a horseshoe-shaped nucleus, while *Trichodina* spp. have a circular body with several rows of cilia.

### 3.5. Prevalence of Endoparasites in Relation to Sex, Size and Seasonal Condition in Nile Tilapia

As presented in Table 4, the infection rate of digenean trematodes was 3% (9 of 300); all of the infected samples were males and the infection was detected only in the summer season. Longer and heavier fish tended to be more susceptible to digenea infection. The infection rate of acanthocephala was 25% (females, 52%; males, 48%); however, infection with acanthocephala was significantly affected by the season, with the highest rate of infection (52%) observed in summer. The overall prevalence of internal protozoa was 11%.

### 3.6. Morphological Characters of the Recovered Endoparasites under Light Microscope

**Figure 2:**
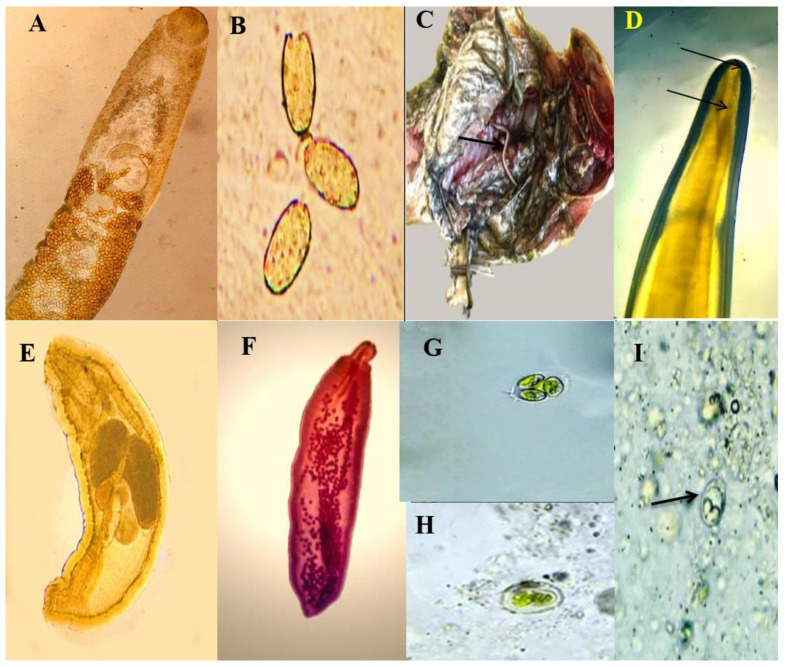
Endoparasites of Nile tilapia. (**A**) *Orientocreadium batrochoides* recovered from intestine ×100; (**B**) egg of *Orientocreadium batrochoides* ×400, small sized egg, yellowish in color and operculated; (**C**) larva of *Contraecum* in the body cavity of Nile tilapia; (**D**) anterior region of *Contracecum* ×100 (arrows show (1) boring tooth and (2) esophagus); (**E**) wet mount of *Acanthosentis tilapiae*. Male recovered from intestine of Nile tilapia ×100; (**F**) female *A. tilapiae* stained with alum carmin; (**G**) sporulated oocyst of *Eimeria* spp. ×1000; (**H**) sporulated oocyst of *Isospora* spp. ×1000; (I) *Myxobolus* spp. ×1000 (arrow shows polar capsule).

As shown in Figure 2, the body of a trematode (*Orientocreadium batrochoides*) is an ellipsoid, with an oral sucker and a ventral sucker. The testicles were tandem in position, posterior to the oval ovary, which is in the middle part of the body. The eggs are numerous, small and operculated. The cuticle of the nematodes (*Contracecum*) was finely serrated anteriorly; their intestinal caeca had a short tail with a retractile tip bearing small spines at the posterior end. The anal opening was subterminal at the end of the body. The Acanthocephala (*Acanthosentis tilapiae*) were characterized by the presence of an anterior retractile proboscis and a tegument with a series of alternative folds. In the males, the testes were unequal in size, while the female reproductive system consisted of a uterus and a uterine duct. Of the protozoa, *Eimeria* spp. sporulated oocysts with four sporocysts were observed, while *Myxobolus* spp. had a polar capsule with polar filaments.

### 3.7. Prevalence of Macroscopic and Microscopic Encysted Metacercariae in Relation to Sex, Size and Seasonal Condition in Nile Tilapia

As presented in Table 5, the overall prevalence of macroscopic encysted metacercariae (*Clinostomum*, and *Euclinostomum*) was 37% (40.5% female and 59.5% male). The total percentage of fish with microscopic encysted metacercariae was 58% (female, 34.5%; male, 65.5%). There were highly significant seasonal variations; the highest infection rates of macroscopic and microscopic metacercariae (59.5% and 36.2%, respectively) were observed in the summer.

### 3.8. Morphological Characters of Macroscopic Encysted Metacercariae (EMC)

**Figure 3:**
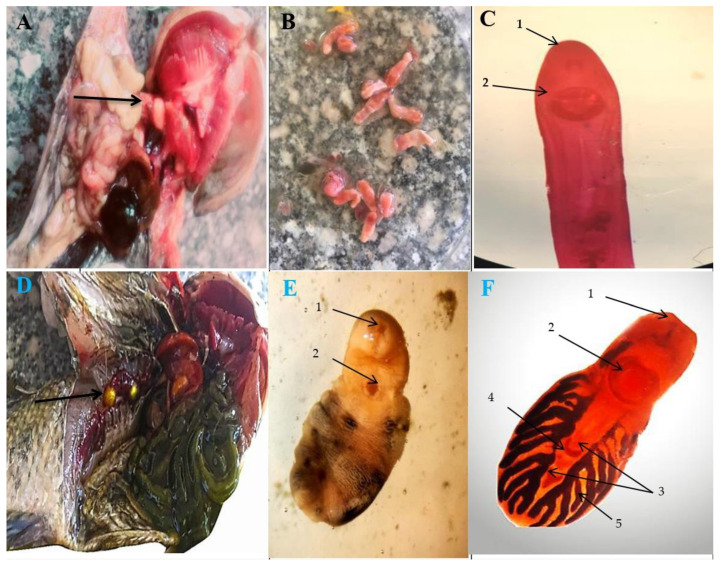
Macroscopic EMC of Nile tilapia. (**A**) Yellow nodule of *Clinostomum* EMC embedded in branchial arch of Nile tilapia (arrow); (**B**) excysted *Clinostomum* spp.; (**C**) *Clinostomum* spp. ×100 stained with acetic acid alum carmine (1—oral sucker, 2—ventral sucker); (**D**) metallic greenish cysts of *Euclinostomum* EMC embedded in kidney tissue; (**E**) excysted *Euclinostomum* spp.×20 (arrows show (1) oral sucker and (2) ventral sucker); (**F**) *Euclinostomum* spp. stained with acetic acid alum carmine ×40 (arrows show (1) oral sucker, (2) ventral sucker, (3) testes (4) ovary and (5) laterally branched ceca.

The encysted metacercariae of *Clinostomum* have flat elongated bodies, with both oral and ventral suckers located in the anterior third (Figure 3). The intestinal caeca branch laterally along the body. The body of *Euclinostomum* has a cylindrical shape with two testes and one ovary.

### 3.9. Morphological Characters of Microscopic Encysted Metacercariae (EMC)

**Figure 4:**
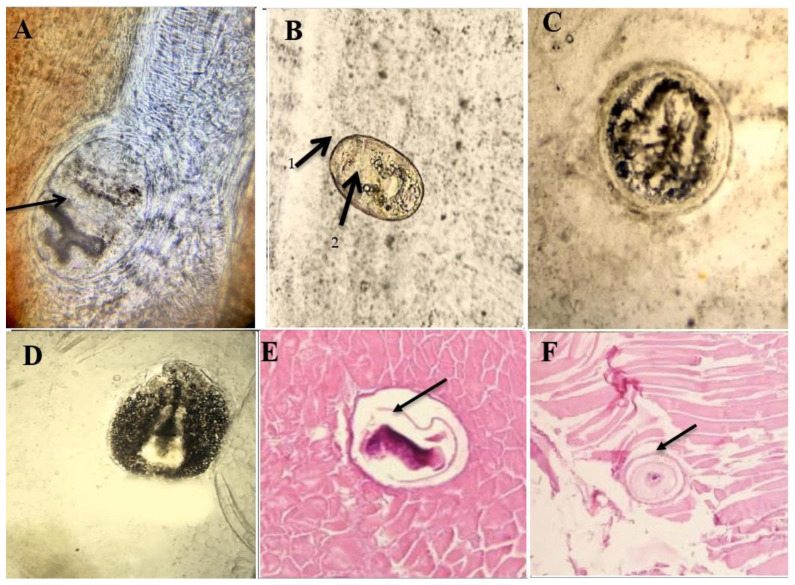
Microscopic EMC of Nile tilapia. (**A**–**D**) Different types of encysted metacercariae in gill filaments of Nile tilapia ×100: (**A**) *Centrocestus formosanus* (arrow shows x-shaped execratory bladder); (**B**) Echinostomatide EMC (arrows show (1) spines and (2) granular execratory bladder); (**C**) *Prohemistomum* EMC in muscle ×100; (**D**) Diplostomatids EMC in muscle ×100; (**E**,**F**) encysted metacercariae in fish muscle (arrows) b(H&E stain) ×100.

As shown in Figure 4, the encysted metacercariae of *Prohemistomum vivax* have a spherically shaped body, are double walled and have a thick outer and inner hyaline wall with pigmented granules. The encysted metacercariae of *Centrocestus* spp. have an elongated elliptical body, an oral sucker surrounded by spines arranged in two rows and an x-shaped excretory bladder. The encysted metacercariae of *Echinostoma* have an oval-shaped body and possess collar spines and corpuscles in their excretory tubes. The encysted metacercariae of *Diplostomum* have an elongated body with a subterminal oval-shaped oral sucker and a subspherical ventral sucker.

### 3.10. Morphological Identification of Worms Obtained through Experimental Infection of Mice with Digested Encysted Metacercariae

**Figure 5:**
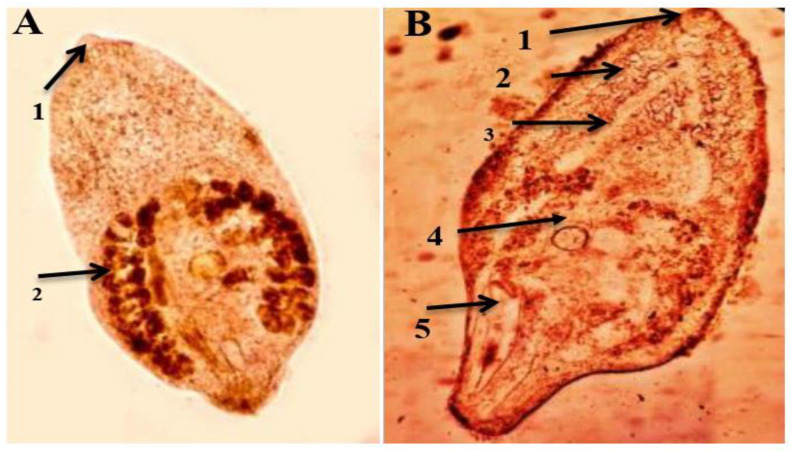
Adult recovered from experimental infection of mice with encysted metacercariae under light microscope. (**A**) *Prohemistomum vivax* adult stage ×100 (1—oral sucker, 2—horseshoe-shapes vitelline glands); (**B**) *Mesostephanus* spp. ×100 (1—oral sucker, 2—horseshoe-shapes vitelline glands, 3—intestinal caeca, 4—testes, 5—cirrus pouch).

**Figure 6:**
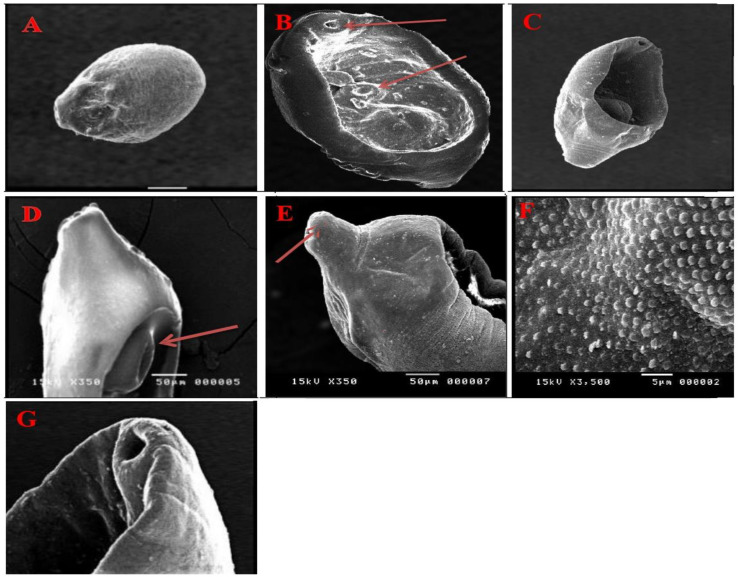
Adult worm recovered from experimental infection of Mice with encysted metcercaria by Scanning Electron Microscope (SEM). (**A**,**B**) *Prohemistomum vivax* (**A**) dorsal view with pyriform or oval shape (**B**) ventral view represented by arrows show oral sucker and ventral sucker. (**C**–**G**) *Mesostephanus* sp. (**C**) ventral view with elongated shape and caudal appendage. (**D**) Posterior end arrow shows tribocytic organ. (**E**) Arrows show caudal appendage and spins. (**F**) spines on tegument (**G**) oral sucker.

As shown in Figure 5 and Figure 6, the adult worms collected from the mice with encysted metacercariae on the seventh day post-infection were identified. Adult worms of *Prohemistomum vivax* were pyriform in shape and with the esophagus bifurcating into two intestinal caeca until reaching the level behind the posterior testes. There was a small round-shaped ovary lateral to the anterior testes and horseshoe-shaped vitelline glands in the middle third of the body. There were two testes large, in tandem position in the posterior half of the worm. *Mesostephanus* spp. is elongated with a caudal appendage and a tegument covered with spines anteriorly. The worm has a subterminal oral sucker, while the ventral sucker occupies the middle part of the body; behind it is a tri-bocytic organ, which is about one-third of the body width and was interpreted as a third sucker-like organ. The pharynx of *Mesostephanus* spp. is well-developed with a simple intestinal caeca. Meanwhile, the testes are ovoid, smooth and oblique in position and located in the posterior half of the body. Both the cirrus pouch and the uterus are well-developed. The folliculated vitellaria are fairly large.

## 4. Discussion

Fish parasites are among the most common infectious agents affecting aquaculture production worldwide, alongside their potential zoonotic threat. The present study collected the baseline information about the occurrence of parasites in Nile tilapia (*Oreochromus niloticus)* in Upper Egypt, along with the main risk factors associated with infection. As mentioned previously, the prevalence rate was 82% (246 out of 300). This result is higher than that recorded in Egypt in a previous work, in 2011 [11], which found a total prevalence rate of 68.8%; in a study in California, USA [43], the prevalence rate was found to be 60%. This high rate of infection in Egypt may be attributed to the variable environmental conditions and increasing pollution, leading to decreased immunity in fish, making them more susceptible to parasitic infection [44]. The total ectoparasites infection rate in the present work was 41%, which is similar to that found in a previous study in Ethiopia (32.29%). In addition, the monogenean infection rate was 31%, which is higher than that recorded in Fayom (Egypt) (1.77%) [45]. Our present results are also higher than several previous works at the international level, including studies in Thailand and Ethiopia, which detected *Dactaylogyrus* spp. in 15% and 4% of the examined fish, respectively [46,47], and an infection rate of external protozoa of 6% and 4% for *Trichodina* and *Icthyophthirius multifiliis*, respectively. This is higher than that recorded in Sharkia [48], where *Trichodina* spp. was identified in 0.02% of the examined fish. In contrast, these results are lower than those recorded in Thailand, where the infection rate with *Trichodina* spp. was 36% [47]. In Indonesia, Kolia et al. (2021) [49] found a *I. multifiliis* infection rate of 15.8%. These differences in the infection rates between studies may be due to ecological differences in the sites from which the fish were collected, as environmental conditions have a significant effect on parasitic infection rates [5,50,51].

The total internal parasitic infection rate was 41% (including trematodes, acanthocephala, nematodes and protozoa), which is similar to that in a previous study in Nigeria, which revealed a prevalence rate of 32.9% [52], and to that in a study by Gebreegziabher et al. (2020) [53] in Ethiopia, which found a rate of 38.6%. On the other hand, our findings are higher than the rate recorded in Fayom (48%) [45]. The prevalence of trematodes (digenea) in the present study was 3%, which was lower than that found in Fayom (10.66%) [45], which might be attributed to feeding behavior [54]. The acanthocephala infection rate was 25%, which was lower than that recorded in Sohag, (35.9%) [55], but higher than that found in other works in Benha (1.5%) [13], Fayom (14%) [45] and the Kafr El-sheikh Governorate (4.2–10.2%) [18,56]. Human cases of acanthocephaliasis are rare in the medical literature; however, there have been a growing number of cases reported in the last ten years (Mathison, et al. 2021) due to an increase in infections in consumed fish. Acanthocephaliasis mainly causes GIT disease.

The nematode infection rate (*Contracecum* spp.) in the present work was 2%, which is similar to that in a previous study [57] in Kenya (2.0%) [58] and another study [25] in Qena (2.3%). In contrast, the present results are lower than those recorded in Kenya (8.9%) [59], Ethiopia (18.5%) [53] and El Minia (50.0%) [42]. The rate of internal protozoa in the present study was 11%, which is lower than that found in a previous study in Sharkia Governorate, Egypt [38], possibly due to the difference in the climatic condition between upper and lower Egypt. In the present study, the infection rate of microscopic encysted metacercariae was 58%, while the macroscopic infection rate was 37%. These results are similar to those of a study in Benha (Egypt) [13], which found a macroscopic EMC rate of 30.5% in Nile tilapia. Another study in El-Minia, Egypt, detected EMC in 50% of the examined fish [42], while in Alexandria, it was found that the microscopic EMC rate was 57.3% [60]. Our results are higher than those mentioned in a previous study in northern Egypt [61], while another study in southern Egypt [18] found a prevalence rate of 29.2%. Our results are lower than those of several previous studies at the national level (Egypt), including in Ismailia (70%), Port Said (78.6%), Sharkia (84.8%), Assiut (78.25%) and Sharkia province (83%) [32,38,62,63,64]. The high prevalence of EMC may be due to the widespread nature of the intermediate host (snails) and the failure of effective control [65].

In the present work, the experimental infection of mice with EMC was performed to reveal the zoonotic trematode species that may be endemic in upper Egypt and that contribute to GIT problems in humans who consume undercooked fish. The worms recovered were *Prohemistomum vivax* and *Mesostephanus* spp. The morphological features of the obtained trematodes were similar to those previously described in several studies in Port Said, Sharkia, Giza and Ismailia, Egypt [16,62,64,65,66]. Regarding the risk factors investigated in this study, sex was found to be a significant risk factor affecting ectoparasites in Nile tilapia. This is similar to a previous study in Ismailia (Egypt) [38]. Concerning size, small fish were more susceptible to parasitic infection compared to larger fish, which is in agreement with a previous study in Giza Governorate (Egypt) [16]. This may be due to the poorly developed immune systems of smaller fish [2,67].

Regarding seasonal variation as a potential factor affecting parasitic infection in Nile tilapia, the highest rate of infection with monogenean trematodes (35.5%) was detected in the winter, with the lowest rate found in autumn (16.1%). The highest rate of infection with external protozoa (40%) was reported in the spring. On other hand, the highest rate of infection with digenean trematodes and acanthocephala (100% and 52%, respectively) was observed in the summer. The highest rate of infection with internal protozoa (36.36%) was detected during the winter. Regarding the available literature, several previous studies found that the highest rate of infection was in the summer, followed by the spring [16,38,42,60,61,62,68]. A study [18] in southern Egypt found the highest prevalence during the spring and the lowest prevalence was in autumn. A study [69] in Cairo detected the highest prevalence in autumn and the lowest in the summer, while a previous work [1] in Kafr El-Sheikh recorded the highest infection rates in autumn and winter. This difference might reflect the reduced feeding activity of fish at low temperatures, thus reducing the chances of infection via copepods [70].

## 5. Conclusions

The present study is one of the few novel studies on fish parasites in Upper Egypt. The study revealed that different parasites are highly prevalent among fresh water fish (Nile tilapia) and have a direct effect on fish health and meat quality and, thus, a direct effect on fish production and the economy. Given the zoonotic potential of these parasites, control measures should be taken into consideration for the prevention of the further spread of these diseases. Furthermore, public health awareness should be raised in the occupational groups dealing with fish to prevent the spread of zoonotic parasites through the consumption of undercooked fish.

## Figures and Tables

**Table 1:** Infection rate in relation to sex, size, weight and seasonal variation in Nile tilapia (*Oreochromis niloticus*).

Variable	Non-Infected (*n* = 54)	Infected (*n* = 246)	*p*-Value
Gender			
Female	15 (28%)	99 (40%)	0.410
Male	39 (72%)	147 (60%)	
Length/cm	17.25 ± 3.9	15.69 ± 2.9	0.113
Weight/gram	117.25 ± 10.9	88.64 ± 16.9	0.024 *
Season			
Winter	24 (44.4%)	51 (21.3%)	0.090
Autumn	19 (35%)	56 (22.7%)	
Spring	4 (7.6%)	71 (28.8%)	
Summer	7 (13%)	68 (27.5%)	

* Significant (*p*-value < 0.05).

**Table 2:** Prevalence of ectoparasites and endoparasites in examined fish.

External Infection (No. Examined = 300)		Internal Infection (No. Examined = 300)	
Parasite	No. Inf. (%)	Parasite	No. Inf. (%)
Monogenic trematodes *Gyrodactylus* *Dactylogrus* *Cichlidogyrus*	15 (5%) 12 (4%) 66 (22%)	Digean trematode *Orientocreadium batrachoides*	9 (3%)
		Nematode *Contracaecum*	6 (2%)
External protozoa *Trichodina* *Icthyophthirius multifiliis*	18 (6%) 12 (4%)	Internal protozoa *Myxobolus* *Isospora* *Eimeria*	6 (2%) 3 (1%) 24 (8%)
		Acanthocephala	75 (25%)
Total	123 (41%)		123 (41%)

**Table 3:** Prevalence of ectoparasites in relation to sex, size and seasonal condition in Nile tilapia fish.

Variable	Non-Infected (*n* = 207)	Infected (*n* = 93)	*p*-Value *	Non-Infected (*n* = 270)	Infected (*n* = 30)	*p*-Value
Parasite	Trematode (Monogeneans)			External Protozoa		
Gender						
Female	66 (31.9%)	48 (51.6%)	=0.060	99 (36.7%)	15 (50%)	<0.001 **
Male	141 (68.1%)	45 (48.4%)		171 (63.3%)	15 (50%)	
Length/cm	16.35 ± 3.4	15.23 ± 2.8	=0.110	16.01 ± 0.35	16.10 ± 0.75	0.7609
Weight/gram	99.32 ± 9.8	83.32 ± 4.9	=0.033 *	93.36 ± 3.57	92.70 ± 1.33	0.4628
Season						
Winter	42 (20.3%)	33 (35.5%)	=0.093	75 (27.7%)	0 (0%)	=0.261
Autumn	60 (29%)	15 (16.1%)		66 (24.5%)	6 (20%)	
Spring	54 (26.1%)	21 (22.6%)		63 (23.3%)	12 (40%)	
Summer	51 (24.6%)	24 (25.8%)		66 (24.5%)	12 (40%)	

The Chi square test was used to compare the difference in infection frequency by sex. An independent t-test was used to compare the mean difference by length and weight. The Monte Carlo exact test was used to compare the difference in frequency by season. * significant; ** highly significant. *p* < 0.05.

**Table 4:** Prevalence of endoparasites in relation to sex, size and seasonal condition in Nile tilapia.

Variable	Non-Infected (*n* = 291)	Infected (*n* = 9)	*p*-Value	Non-Infected (*n* = 225)	Infected (*n* = 75)	*p*-Value	Non-Infected (*n* = 267)	Infected (*n* = 33)	*p*-Value
Parasite	Trematode (Digeneans)			Acanthocephalan			Internal protozoa		
Gender									
Female	114 (39.2%)	0 (0%)	0.169	75 (33.3%)	39 (52%)	0.096	99 (37%)	15 (45.5%)	0.307
Male	177 (60.8%)	9(100%)		150 (66.7%)	36 (48%)		168 (62.9%)	18 (54.6%)	
Length/cm	15.90 ± 3.2	19.33 ± 2.5	0.065	15.95 ± 3.4	16.16 ± 2.7	0.777	16.27 ± 3.2	15.01 ± 3.1	0.101
Weight/gram	93.89 ± 5.1	109.67 ± 4.8	0.059	96.56 ± 8.5	87.76 ± 9.1	0.137	96.03 ± 5.1	88.10 ± 3.4	0.346
Season									
Winter	75 (25.8%)	0 (0%)	0.380	75 (33.3%)	0 (0%)	<0.001 **	63 (23.59%)	12(36.36%)	0.392
Autumn	75 (25.8%)	0 (0%)		69 (30.7%)	6 (8%)		66 (24.7%)	9 (27.2%)	
Spring	75 (25.8%)	0 (0%)		45 (20%)	30 (40%)		69 (25.8%)	6 (18.1%)	
Summer	66 (22.6%)	9 (100%)		36 (16%)	39 (52%)		69 (25.8%)	6 (18.8%)	

The Chi square test was used to compare the difference in frequency by sex. An independent t-test was used to compare the means difference by length and weight. The Monte Carlo Exact test was used to compare the difference in frequency by season. ** highly significant. *p* < 0.05.

**Table 5:** Prevalence of macroscopic and microscopic encysted metacercariae in relation to sex, size and seasonal condition.

Variable	Non-Infected (*n* = 189)	Infected (*n* = 111)	*p*-Value	Non-Infected (*n* = 126)	Infected (*n* = 174)	*p*-Value
Parasite	Macroscopic EMC			Microscopic EMC		
Gender						
Female	69 (36.5%)	45 (40.5%)	0.688	54 (42.9%)	60 (34.5%)	0.394
Male	120 (63.5%)	66 (59.5%)		72 (57.1%)	114 (65.5%)	
Length/cm	15.87 ± 3.6	16.22 ± 2.5	0.577	16.33 ± 3.7	16.76 ± 3.3	0.385
Weight/gram	97.19 ± 5.7	89.54 ± 3.6	0.224	102.28 ± 6.7	88.02 ± 3.3	0.074
Season						
Winter	75 (39.7%)	0 (0%)	<0.001 **	42 (33.3%)	33 (19%)	0.002 *
Autumn	66 (34.9%)	9 (8.1%)		48 (38.1%)	27 (15.5%)	
Spring	39 (20.6%)	36 (32.4%)		24 (19%)	51 (29.3%)	
Summer	9 (4.8%)	66 (59.5%)		12 (9.5%)	63 (36.2%)	

The Chi square test was used to compare the difference in frequency by sex. An independent t-test was used to compare mean difference by length and weight. The Monte Carlo exact test was used to compare the difference in frequency by season. * significant; ** highly significant. *p* < 0.05.

## Data Availability

The data presented in this study are available on request from the corresponding author.
